# Anterior Redisplacement After Intramedullary Nail Fixation for Trochanteric Femoral Fractures: Incidence and Risk Factors in 598 Older Patients

**DOI:** 10.3390/jcm14155557

**Published:** 2025-08-06

**Authors:** Hironori Kuroda, Suguru Yokoo, Yukimasa Okada, Junya Kondo, Koji Sakagami, Takahiko Ichikawa, Keiya Yamana, Chuji Terada

**Affiliations:** 1 Department of Orthopaedic Surgery, Fukuyama City Hospital, Hiroshima 721-8511, Japan; hironori-kuroda@nms.ac.jp (H.K.);; 2 Shock and Trauma Center, Nippon Medical School, Chiba Hokusoh Hospital, Inzai 270-1694, Japan

**Keywords:** hip fractures, aged, femoral fractures, bone nails, fracture fixation, internal, reoperation, treatment failure, postoperative complications

## Abstract

**Background/Objectives:** Anterior redisplacement, defined as a postoperative anterior shift of the distal fragment despite intraoperative reduction, is occasionally observed after cephalomedullary nailing for trochanteric femoral fractures. However, its incidence and associated risk factors remain unclear. This study aimed to determine the incidence of anterior redisplacement following intramedullary nail fixation in geriatric trochanteric fractures, and to identify independent risk factors. **Methods:** This study retrospectively reviewed data from 598 consecutive hips in 577 patients (aged ≥65 years) who underwent intramedullary nail fixation for trochanteric fractures at a single center (2012–2023). Sagittal reduction on the lateral radiographic view was classified as posterior, anatomical, or anterior according to the position of the distal fragment, and was recorded preoperatively and postoperatively. Anterior redisplacement, the primary outcome, was defined as a change in alignment from a posterior or anatomical position postoperatively to an anterior position on any subsequent follow-up radiograph. Independent risk factors were identified by logistic regression. **Results:** Among the 543 hips reduced posteriorly (*n* = 204) or anatomically (*n* = 339), anterior redisplacement occurred in 73 (13.4%). The incidence of anterior redisplacement was significantly higher following anatomical compared to posterior reduction (19.5% vs. 3.4%; *p* < 0.001), and also higher in fractures that were anteriorly aligned preoperatively (18.0%) compared to anatomical (8.5%; *p* < 0.01) and posterior (6.2%; *p* < 0.01) alignment. Multivariate analysis revealed two independent predictors: preoperative anterior alignment (odds ratio [OR] 1.87, 95% confidence interval [CI] 1.24–2.81; *p* = 0.003) and postoperative anatomical (vs. posterior) reduction (OR 6.49, 95% CI 2.92–14.44; *p* < 0.001). Age, sex, Arbeitsgemeinschaft für Osteosynthesefragen/Orthopaedic Trauma Association classification, Evans–Jensen classification, nail length, and canal-filling ratio were not associated with redisplacement. No lag-screw cutout occurred during the follow-up. **Conclusions:** Anterior redisplacement occurred in one of seven geriatric trochanteric fractures despite apparently satisfactory fixation. An anatomical sagittal reduction—traditionally considered “ideal”—increases the risk more than sixfold, whereas a deliberate posterior-buttress is protective. Unlike patient-related risk factors, sagittal reduction is under the surgeon’s control. The study findings provide evidence that choosing a slight posterior bias can significantly improve stability.

## 1. Introduction

Worldwide, intertrochanteric fractures account for roughly half of the 6–7 million hip fractures projected annually by 2050. Most occur in adults aged ≥65 years and are associated with 30-day and 1-year mortality rates of 10% and 20%–30%, respectively [1]. Recent Japanese epidemiological data demonstrate a persistent rise in proximal femoral fractures, with 2005–2014 surveillance documenting a steadily increasing incidence [2]. A 35-year prefectural cohort study further reported 3369 hip fractures in 2020 alongside a pronounced rebound in individuals aged ≥90 years [3]. The resulting loss of independent mobility increases the institutional care demand and generates direct medical costs exceeding USD 20 billion annually in high-income countries [4]. A more recent European report showed that fragility fractures in the EU27+2 region incurred direct healthcare costs of EUR 56.9 billion in 2019—an increase of approximately USD 2000–5000 per case compared with earlier estimates [5].

Failed cephalomedullary fixation—particularly lag-screw or helical-blade cutout—remains the most devastating mechanical complication, requiring revision arthroplasty in up to 7% of cases [6]. In a recent multicenter nested case–control study (*n* = 2327), Inui et al. reported that anterior malreduction—defined as anterior displacement of the distal fragment on postoperative oblique–lateral radiographs—increased the risk of cutout independently of the tip–apex distance [7]. Although the tip–apex distance is a well-established predictor of cutout, their findings shed new light on the independent effect of anterior malreduction, emphasizing its clinical relevance.

Especially in older adults, closed reduction is generally preferred to minimize surgical invasiveness. Moreover, recent studies suggest that closed reduction may also attenuate postoperative sterile inflammatory responses [8]. However, achieving satisfactory sagittal alignment through closed methods alone can be technically challenging. Numerous intraoperative techniques have been developed to assist sagittal reduction in trochanteric fractures, including reduction tools, percutaneous pins, and clamp-based methods. These approaches are particularly useful when satisfactory alignment cannot be achieved by closed manipulation alone [9,10,11,12].

Despite apparently acceptable intraoperative images, clinicians continue to observe anterior redisplacement on early follow-up radiographs [13]. This phenomenon indicates that static malreduction explains only part of the failure mechanism; fracture segments may move secondarily through lag-screw telescoping or posterior sagging before the cortical buttress engages [14,15]. However, the incidence, predictors, and clinical consequences of this dynamic instability have not been quantified in a large cohort.

We hypothesized that fractures initially presenting with an anterior alignment—or reduced to a neutral (anatomical) rather than posterior-buttress position—would exhibit higher rates of anterior redisplacement and, consequently, greater mechanical instability. To test this hypothesis, the position of the bone fragments was evaluated at three distinct time points—preoperatively, immediately postoperatively, and at follow-up—allowing us to identify any anterior redisplacement over time.

## 2. Materials and Methods

### 2.1. Ethics

The study was conducted according to the guidelines of the Declaration of Helsinki and approved by the Institutional Review Board of Fukuyama City Hospital (Approval number 846 dated 29 November 2024). The requirement for written informed consent was waived by the IRB; instead, participants or their guardians were informed about the study via an opt-out notice posted on our hospital website and displayed within the hospital. Data were securely stored in an encrypted hospital database, accessible only to authorized researchers.

### 2.2. Study Design, Setting, and Participants

**Figure 1:**
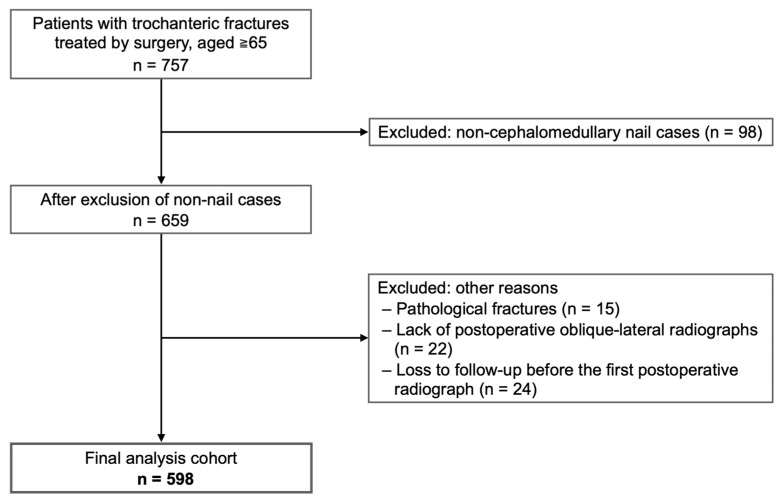
Flow diagram of patient selection.

This single-center, retrospective cohort study included 598 hips from 577 patients aged ≥65 years who underwent intramedullary nail fixation for trochanteric femoral fractures at our institution between January 2012 and December 2023 (Figure 1). The primary objectives were to (i) determine the incidence of anterior redisplacement, (ii) identify preoperative and postoperative risk factors, and (iii) clarify its association with implant failure.

The exclusion criteria were as follows: (1) pathological fractures, (2) lack of postoperative oblique-lateral radiographs, and (3) loss to follow-up before the first postoperative radiograph was taken. After exclusion, 598 hips of 577 patients constituted the final study cohort. Twenty-one patients sustained metachronous contralateral fractures, i.e., the second hip fracture occurred at a different time and required a separate admission. Each patient contributed two hips, which were analyzed as independent observations because the study focused on hip-specific factors. To analyze anterior redisplacement, we stratified the data by postoperative reduction subtype. As anterior redisplacement was defined as a shift from a posterior or anatomical to an anterior position, hips that were already in an anterior position postoperatively were not eligible for this analysis. In accordance with our institution’s protocol, patients were transferred to a rehabilitation facility approximately 2 weeks after surgery, returned for clinical and radiographic assessment at 3 months postoperatively, and were followed-up at 6 and 12 months.

### 2.3. Surgical Technique

All procedures were performed with the patient in the supine position on a traction table under C-arm fluoroscopic guidance. When closed reduction proved difficult, a small lateral incision was made to allow open the reduction of the fracture fragments. Then, an antegrade cephalomedullary nail was inserted through a small proximal window at the apex of the greater trochanter. Detailed implant information is provided in Supplementary Table S1.

### 2.4. Radiographic Classification

Standard anteroposterior and oblique-lateral radiographs of the hip were obtained before surgery, immediately after surgery, at 1 week after surgery, and at routine follow-up visits as determined by the treating physician.

**Figure 2:**
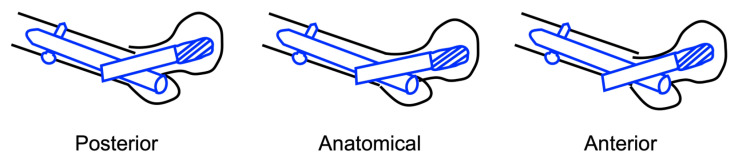
Schematic of the sagittal reduction subtypes on oblique-lateral radiographs.

Sagittal reduction on the oblique-lateral radiographic view was classified into three subtypes (Figure 2):

Posterior—the distal fragment lies posterior to the proximal fragment;

Anatomical—the cortices of the two fragments are co-linear;

Anterior—the anterior cortex of the distal fragment lies anterior to the anterior cortex of the proximal fragment.

### 2.5. Outcomes

The primary outcome was anterior redisplacement, defined as the postoperative migration of a distal fracture segment that had been reduced in an anatomical or posterior position to an anterior position on any follow-up radiograph. The secondary outcome was lag-screw cutout; however, no cutout events occurred during follow-up. Therefore, this endpoint is described but not formally analyzed.

### 2.6. Covariates

The following potential confounders were extracted from the medical records and radiographs: age, sex, comorbidities, Arbeitsgemeinschaft für Osteosynthesefragen/Orthopaedic Trauma Association (AO/OTA) classification (31A1/A2/A3) [16], Evans–Jensen classification (Ⅰ–Ⅴ) [17], nail length (short, <230 mm; middle, 230–260 mm; and long >260 mm), canal-filling ratio (defined as the ratio of the nail diameter to the canal diameter measured on both anteroposterior and oblique-lateral radiographs—at 1 cm proximal to the nail tip for short nails and at the narrowest point of the canal for middle and long nails), and the preoperative sagittal subtype described above.

### 2.7. Statistical Analysis

Continuous variables were tested for normality using the Shapiro–Wilk test. Continuous variables are presented as mean ± standard deviation and were compared through Student’s *t*-test or Welch’s *t*-test, depending on the equality of variances. Categorical variables, which were compared by Fisher’s exact test, are reported as counts with percentages. Variables associated with anterior redisplacement in the univariate analysis (*p* < 0.05) were simultaneously entered into a multivariate logistic regression model. The results are expressed as odds ratios (ORs) with 95% confidence intervals (CIs). Model calibration was assessed using the Hosmer–Lemeshow goodness-of-fit test. A two-sided *p*-value < 0.05 was considered significant. All analyses were performed using EZR (Easy R) version 2.73 (Saitama Medical Center, Jichi Medical University, Saitama, Japan), which is a graphical user interface for R (The R Foundation for Statistical Computing, Vienna, Austria).

## 3. Results

### 3.1. Patient Demographics

During the study period, a total of 598 hips (mean age, 85.6 ± 7.6 years; 444 women and 154 men) underwent intramedullary nail fixation for trochanteric femoral fractures. Baseline comorbidities included cardiovascular disease in 197 hips, renal disease in 96, pulmonary disease in 65, cerebrovascular disease in 123, and dementia in 151 (Table 1).

Various cephalomedullary nails were used, such as InterTAN^®^ (*n* = 302; Smith & Nephew, Memphis, TN, USA), IPT^®^ (*n* = 112; HOMS, Tokyo, Japan), PFNA^®^ (*n* = 109; DePuy Synthes, Zuchwil, Switzerland), and others. Details of all implant types and manufacturers are provided in Table S1.

The subtypes of the preoperative sagittal alignment were posterior in 109 hips (18.2%), anatomical in 151 (25.2%), and anterior in 338 (56.5%).

### 3.2. Incidence of Anterior Redisplacement

Among all 598 fractures, 204 (34.1%), 339 (56.7%), and 55 (9.1%) were reduced to posterior, anatomical, and anterior positions, respectively, on the postoperative radiograph. Given that fractures that had already reduced anteriorly could not translate further anteriorly, these 55 hips were excluded, leaving 543 evaluable hips for the redisplacement analysis.

Among the 543 hips analyzed, posterior reduction was achieved significantly more often in female patients, in fractures classified as more complex by the AO and Evans–Jensen systems, and in cases treated with middle or long intramedullary nails (Table 2).

Overall, anterior redisplacement occurred in 73 of 543 (13.4%) hips. No significant associations were found between anterior redisplacement and age, sex, AO/OTA, Evans–Jensen classification, nail length, or filling ratio (all *p* > 0.05; Table 3).

**Figure 3:**
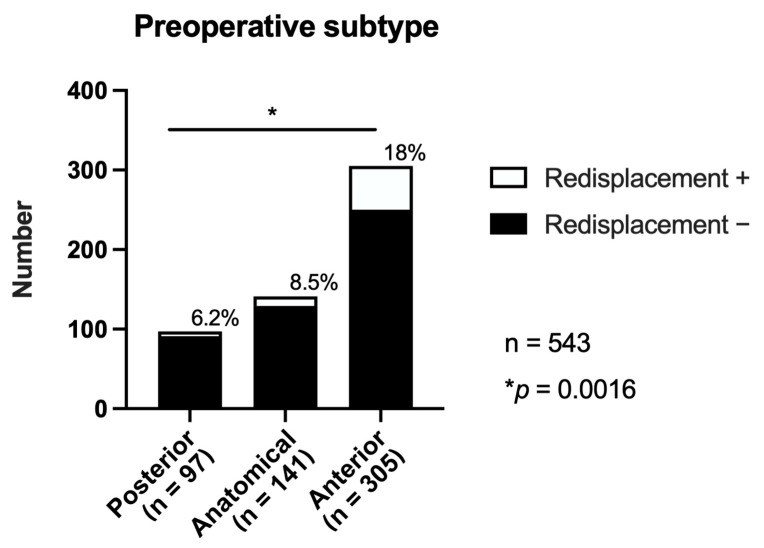
Incidence of anterior redisplacement according to the preoperative alignment subtype.

Preoperative sagittal alignment influenced the risk of redisplacement (Figure 3, Table 4). The incidence rates were 6.2% (6/97), 8.5% (12/141), and 18.0% (55/305) in patients with preoperative posterior, anatomical, and anterior subtypes, respectively (overall, *p* = 0.0016; pairwise *p* < 0.01 for posterior vs. anterior and anatomical vs. anterior).

**Figure 4:**
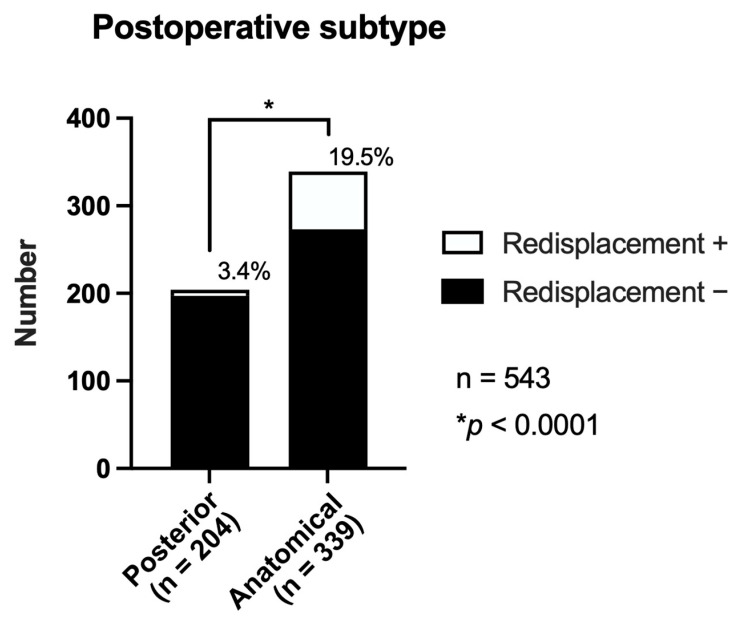
Incidence of anterior redisplacement according to the postoperative reduction subtype.

Postoperative sagittal reduction showed a strong association with anterior redisplacement (Figure 4, Table 5). Anterior redisplacement was significantly more frequent in anatomical reduction than in posteriorly reduced hips (19.5% [66/339] vs. 3.4% [7/204]; *p* < 0.0001).

The white portions of the bars represent cases with anterior redisplacement, whereas the black portions represent cases without redisplacement. The percentage above each bar indicates the incidence of redisplacement in that group—6.2%, 8.5%, and 18.0% in the posteriorly, anatomically, and anteriorly aligned groups, respectively. The overall comparison across subtypes was significant (* *p* = 0.0016, Fisher’s exact test). The total number of cases *n* = 543.

Incidence of anterior redisplacement according to the postoperative reduction subtype. The anatomically reduced group exhibited a significantly higher incidence of redisplacement (19.5%) than the posteriorly reduced group (3.4%) (*p* < 0.0001).

The incidence was significantly higher in the anatomically reduced group (19.5%) than in the posteriorly reduced group (3.4%). Cases with the postoperative anterior subtype (*n* = 55) were excluded from this analysis.

### 3.3. Multivariate Analysis

Multivariate logistic regression identified two independent risk factors for anterior redisplacement (Table 6), namely, preoperative anterior position (vs. non-anterior) with an OR of 1.87 (95% CI: 1.24–2.81, *p* = 0.003) and postoperative anatomical reduction (vs. posterior) with an OR 6.49 (95% CI: 2.92–14.44, *p* < 0.001). The model showed acceptable calibration (Hosmer–Lemeshow, χ^2^ = 7.265, *p* = 0.123), with a Nagelkerke R^2^ of 0.134, and correctly classified 86.5% of the cases.

These results indicate that an anteriorly displaced fracture pattern before surgery and an anatomical (rather than posterior) sagittal reduction following intramedullary nailing markedly increase the risk of anterior redisplacement during follow-up.

Reference categories: preoperative position = non-anterior (anatomical/posterior); postoperative reduction = posterior reduction.

In the multivariate logistic regression (Table 6), both preoperative anterior position and postoperative anatomical reduction were independent predictors of anterior redisplacement. Specifically, a preoperative anterior position was associated with a 1.87-fold increase in the odds of redisplacement (OR 1.87, 95% CI 1.24–2.81; *p* = 0.003), and anatomical postoperative reduction conferred a 6.49-fold higher OR than posterior reduction (OR 6.49, 95% CI 2.92–14.44; *p* < 0.001).

## 4. Discussion

### 4.1. Principal Findings

In this large, single-center cohort of 598 hips from older patients who underwent intramedullary nailing for the treatment of trochanteric femoral fractures, anterior redisplacement occurred in 13.4% of the 543 hips that had been reduced in either an anatomical or the posterior position. Multivariate analysis identified two independent and clinically actionable risk factors—a preoperative anterior fracture alignment almost doubled the odds of redisplacement (OR: 1.87), and a postoperative anatomical reduction increased the odds more than sixfold compared with posterior reduction (OR: 6.49) (Table 6). Neither fracture morphology (AO/OTA and Evans–Jensen classification) nor implant-related variables (nail length and filling ratio) were associated with redisplacement (Table 3). Taken together, these findings indicate that sagittal alignment—particularly the surgeon-controlled achievement of a slight posterior reduction—outweighs implant choice and fracture complexity as the dominant, modifiable determinant of postoperative stability.

Compared with hips reduced to an anatomical position, posterior reduction on postoperative radiographs was more frequently observed in female patients, in those with more complex fracture patterns, and in those treated using middle or long nails (Table 2). These patterns largely reflect the surgeon’s preference; posterior alignment was deliberately selected for female patients with osteoporosis and those with unstable fractures. This selection bias may have influenced the final outcomes because posterior reduction was more likely achieved in cases where postoperative redisplacement was already of concern.

### 4.2. Pathophysiological Interpretation

Postoperative anterior redisplacement occurs when the distal shaft fragment does not lie posterior to the head–neck fragment, leaving the anteromedial cortex unsupported. Under axial loading, this produces a sagittal-swing moment that drives the proximal fragment further into flexion. Once the lag-screw telescopes, secondary sliding amplifies the displacement and shortens the neck [13,18,19]. In contrast, placing the head–neck fragment slightly posterior (≤1 cortical thickness) to the shaft establishes an anteromedial buttress that converts shear into compression and allows the sharing of loads between the bone and the nail. Biomechanical tests have shown that compared with an intramedullary (anterior) reduction, this “extramedullary” or “positive medial cortical support” configuration halves telescoping and reduces nail migration [20,21]. In the present cohort, such posterior over-reduction lowered the odds of redisplacement sixfold (OR: 6.49).

To our knowledge, this study is the first to investigate the relationship between preoperative displacement and the postoperative loss of reduction. Patients presenting with anterior shift of the distal segment preoperatively were more likely to experience postoperative redisplacement, even when anatomical or posterior reduction was achieved (Table 4, Table S2). This finding may suggest an inherent instability in fracture patterns that initially present with anterior displacement.

### 4.3. Comparison with Previous Studies

Most studies have focused on coronal alignment and the tip–apex distance, with relatively few large-scale analyses of sagittal reduction and postoperative redisplacement [7,22]. Biomechanical and small clinical reports have shown that anterior malreduction increases the risk of lag-screw cutout and implant sliding, whereas positive medial cortical support limits impaction and pain [13,19,20]. Although no cutout events occurred in the study cohort and functional outcomes were not assessed systematically, these data collectively suggest that avoiding anterior redisplacement remains a prudent surgical objective.

More recent series have identified dynamic risk factors for redisplacement—comminution at the greater trochanter and low lateral canal-filling ratio [23]; however, in our cohort, the filling ratio was not predictive, and Evans–Jensen type III–Ⅴ fractures did not exhibit greater instability (Table 3). This highlights the primacy of anteromedial buttress over fracture morphology. However, posterior reduction was more often applied in cases with presumed greater instability, which may have influenced these results (Table 2).

Several recent studies have employed three-dimensional computed tomography to characterize sagittal fragment alignment and cortical buttress formation more precisely [14,23,24], an approach not employed in the present radiograph-based analysis.

By modeling both preoperative and postoperative sagittal alignment within a single logistic framework in the largest uniform radiograph-based cohort to date (*n* = 598), this study clarified their independent and additive contributions to postoperative stability. This study extends this literature in three ways, by (i) evaluating dynamic redisplacement rather than static malreduction, capturing fractures that were acceptable intraoperatively but destabilized during early loading; (ii) modeling preoperative and postoperative alignment within the same logistic framework, clarifying their independent and additive contributions to instability; and (iii) analyzing the largest single-institution cohort to date (*n* = 598) with uniform imaging and follow-up, thereby narrowing the CIs around the effect estimates. Importantly, the fact that Evans–Jensen type III–V fractures did not exhibit higher redisplacement rates (Table 3) underscores the critical importance of support at the thick anteromedial cortex, irrespective of posterior comminution.

### 4.4. Clinical Implications: Why a Deliberate Posterior Reduction Deserves Routine Consideration

The present findings, together with converging biomechanical and clinical evidence [7,18,19,25], challenge the traditional goal of an anatomically flush sagittal cortex and support a controlled posterior offset, defined as leaving the head–neck fragment ≤1 cortical thickness posterior to the shaft. Leaving the head–neck fragment slightly posterior to the shaft (i) transforms shear into compressive loading across the buttress, (ii) limits telescoping to <2 mm, thus preventing the 5–10 mm of uncontrolled shortening often seen after “anatomical” reductions, and (iii) mitigates varus drift and lag-screw cutout risk. Importantly, this target position is easy to visualize intraoperatively; a 30° oblique-lateral radiographic view should show the distal anteromedial cortex overlapping the proximal spike [26]. When closed manipulation yields a flush or anterior cortex, surgeons should favor a deliberate posterior over-reduction—achievable with a small anteriorly directed elevator—rather than additional traction or nail exchange. Incorporating an item such as “anteromedial cortical buttress achieved (yes/no)” into intraoperative checklists, alongside the tip–apex distance and neck–shaft angle, may facilitate the consistent adoption of this technique.

Although implant failure was rare in our cohort, anterior redisplacement has been linked in previous studies to increased hip pain, delayed weight-bearing, and a higher risk of lag-screw cut-out [7,14,19]. Because posterior reduction is a simple, low-cost maneuver that can be adopted intraoperatively, minimizing redisplacement may still yield meaningful patient benefits even when catastrophic failures are uncommon.

### 4.5. Strengths and Limitations

The strengths are related to the study being the largest single-center series to date, the use of uniform surgical techniques, blinded radiographic adjudication, and multivariable modeling that separates preoperative and postoperative alignment.

Limitations are intrinsic to the study design, and include the following:

1. Single-institution, retrospective cohort—practice patterns may differ elsewhere; selection and information bias cannot be fully excluded;
2. Follow-up heterogeneity—although 92% of patients had ≥3 months of imaging data, late attrition may underestimate very delayed redisplacement;
3. Unmeasured confounders—bone density, surgeon experience, and rehabilitation protocols were not captured; each could influence stability.
  These caveats tamper the generalizability of our numeric risk estimates, but not the biomechanical principle that the anteromedial buttress matters;
4. Clinical outcomes—functional or symptomatic endpoints—including walking capacity, pain, and quality-of-life scores—were not systematically captured in this retrospective cohort. Although lag-screw cut-out was predefined as a secondary endpoint, no such failures were observed, and postoperative lag-screw telescoping was not quantified. Prospective studies that collect patient-reported outcome measures and correlate them with the degree of anterior redisplacement are therefore warranted.

### 4.6. Future Directions

Prospective, multicenter trials should examine deliberate posterior-biased reduction versus anatomical reduction with standardized implants, rehabilitation, and longer (>12 months) follow-up. Embedding intraoperative motion analysis—for example, optical tracking of fragment drift between reduction and wound closure—could identify micro-instability invisible on two-dimensional fluoroscopy. Finally, finite-element and cadaver models integrating patient-specific bone density may clarify how much posterior offset is optimal for different fracture patterns.

## 5. Conclusions

Preoperative anterior fracture alignment and postoperative anatomical reduction are independent, clinically significant risk factors for anterior redisplacement following intramedullary nailing of trochanteric femoral fractures in older patients. Achieving—or deliberately leaving—a slight posterior reduction offers a simple, readily modifiable strategy to mitigate this complication, and may translate into better functional outcomes and fewer implant failures.

## Figures and Tables

**Table 1:** Baseline characteristics of the study population (*n* = 598).

Variable	
Number of cases	598
Sex (female/male)	444/154
Age, years (mean ± SD)	85.8 ± 7.5
Comorbidities	
Cardiovascular disease	197
Renal disease	96
Pulmonary disease	65
Cerebrovascular disease	123
Dementia	151
AO/OTA classification	
A1	259
A2	268
A3	71
Evans–Jensen classification	
Ⅰ	23
Ⅱ	58
Ⅲ	217
Ⅳ	13
Ⅴ	287
Nail length	
Short (<230 mm)	435
Middle (230 to <260 mm)	98
Long (>260 mm)	65
Preoperative subtype	
Posterior	109
Anatomical	151
Anterior	338
Filling ratio (mean ± SD)	
Anteroposterior view	0.80 ± 0.10
Oblique-lateral view	0.70 ± 0.10

Abbreviations: SD, standard deviation; AO/OTA, Arbeitsgemeinschaft für Osteosynthesefragen/Orthopaedic Trauma Association.

**Table 2:** Characteristics of patients showing posterior or anatomical reductions in postoperative radiograph.

Variable	All (*n* = 543)	Posterior (*n* = 204)	Anatomical (*n* = 339)	*p*-Value
Sex				0.02
Female	411	166	245	
Male	132	38	94	
Age, years (mean ± SD)	85.8 ± 7.6	86.2 ± 7.6	85.5 ± 7.6	0.29
Comorbidities				
Cardiovascular disease	181	69	112	0.85
Renal disease	85	33	52	0.81
Pulmonary disease	62	25	37	0.68
Cerebrovascular disease	113	45	68	0.59
Dementia	141	49	92	0.48
AO/OTA classification				0.02
A1	231	71	160	
A2	249	105	144	
A3	63	28	35	
Evans–Jensen classification				0.001
Ⅰ	23	2	21	
Ⅱ	54	14	40	
Ⅲ	185	65	120	
Ⅳ	12	5	7	
Ⅴ	269	118	151	
Nail length				0.001
Short (<230 mm)	390	130	260	
Middle (230 to <260 mm)	93	50	43	
Long (>260 mm)	60	24	36	
Filling ratio (mean ± SD)				
Anteroposterior view	0.80 ± 0.10	0.80 ± 0.10	0.81 ± 0.10	0.46
Oblique-lateral view	0.70 ± 0.10	0.71 ± 0.10	0.70 ± 0.10	0.09

Abbreviations: SD, standard deviation; AO/OTA, Arbeitsgemeinschaft für Osteosynthesefragen/Orthopaedic Trauma Association. Patient characteristics stratified by postoperative sagittal reduction. Posterior reduction was more commonly observed in female patients, who are presumed to have more osteoporotic bone, and in those with more complex fracture types based on the AO/OTA and Evans–Jensen classifications. Additionally, longer nails (middle or long) were more frequently used in the posterior group, which may reflect the surgeon’s intraoperative assessment of greater mechanical instability.

**Table 3:** Characteristics of patients with anterior redisplacement after surgery.

Variable	All (*n* = 543)	Anterior Redisplacement (*n* = 73)	No Redisplacement (*n* = 470)	*p*-Value
Sex				0.77
Female	411	54	357	
Male	132	19	113	
Age, years (mean ± SD)	85.8 ± 7.6	85.4 ± 7.6	85.8 ± 7.6	0.68
Comorbidities				
Cardiovascular disease	181	19	162	0.18
Renal disease	85	13	72	0.60
Pulmonary disease	62	8	54	1.0
Cerebrovascular disease	113	16	97	0.76
Dementia	141	17	124	0.67
AO/OTA classification				1.00
A1	231	31	200	
A2	249	34	215	
A3	63	8	55	
Evans–Jensen classification				0.26
Ⅰ	23	1	22	
Ⅱ	54	5	49	
Ⅲ	185	22	163	
Ⅳ	12	3	9	
Ⅴ	269	42	227	
Evans–Jensen classification				0.12
Ⅰ + Ⅱ	77	6	71	
Ⅲ − Ⅴ	469	67	399	
Nail length				0.74
Short (<230 mm)	390	55	335	
Middle (230 to < 260 mm)	93	10	83	
Long (>260 mm)	60	8	52	
Filling ratio (mean ± SD)				
Anteroposterior view	0.80 ± 0.10	0.80 ± 0.10	0.81 ± 0.09	0.67
Oblique-lateral view	0.70 ± 0.10	0.70 ± 0.10	0.69 ± 0.09	0.39

Abbreviations: SD, standard deviation; AO/OTA, Arbeitsgemeinschaft für Osteosynthesefragen/Orthopaedic Trauma Association. No significant associations were observed for age, comorbidities, fracture classification, or implant characteristics (all *p* > 0.05).

**Table 4:** Incidence of anterior redisplacement according to the preoperative alignment subtype.

Preoperative Subtype	Anterior Translation (+)	Anterior Translation (−)	Total	Incidence (%)	*p*-Value
Posterior	6	91	97	6.2%	-
Anatomical	12	129	141	8.5%	-
Anterior	55	250	305	18.0%	0.0016
Total	73	470	543	13.4%	

The incidence of anterior redisplacement varied significantly by preoperative sagittal alignment, occurring in 6.2%, 8.5%, and 18.0% of hips with posterior, anatomical, and anterior subtypes, respectively (overall *p* = 0.0005; pairwise *p* < 0.01 for posterior vs. anterior and anatomical vs. anterior).

**Table 5:** Incidence of anterior redisplacement according to the postoperative reduction subtype.

Preoperative Subtype	Anterior Translation (+)	Anterior Translation (−)	Total	Incidence (%)	*p*-Value
Posterior	7	197	204	3.4%	<0.0001
Anatomical	66	273	339	19.5%	-
Total	73	470	543	13.4%	

**Table 6:** Multivariate logistic regression analysis of the risk factors for anterior redisplacement.

Variable	OR (95% CI)	*p*-Value
Preoperative anterior position (vs. non-anterior)	1.87 (1.24–2.81)	0.003
Postoperative anatomical reduction (vs. posterior)	6.49 (2.92–14.44)	<0.0001

Abbreviations: OR, odds ratio; CI, confidence interval.

## Data Availability

Due to ethical considerations, we are unable to make the full dataset publicly available. However, we are open to discussing requests for anonymized data from qualified researchers under appropriate agreements.

## Supplementary Materials

The following supporting information can be downloaded at: https://www.mdpi.com/article/10.3390/jcm14155557/s1, Table S1. Cephalomedullary nail implants used in the study population: device names, manufacturers, and number of cases. Table S2. Preoperative and postoperative subtypes with the number and percentage of anterior redisplacement cases.
