# Supplementary material for: Anterior Redisplacement After Intramedullary Nail Fixation for Trochanteric Femoral Fractures: Incidence and Risk Factors in 598 Older Patients
Source: J Clin Med. 2025 Aug 6;14(15):5557. doi: 10.3390/jcm14155557 (PMC12346959; doi:10.3390/jcm14155557)
Supplement: Supplementary file 1 [file jcm-14-05557-s001.zip › Supplementary_Table_S1.pdf]

Table S1. Cephalomedullary nail implants used in the study population: device names, manufacturers, and number of cases

**Implant**	**Number of Cases**
InterTAN® (Smith & Nephew, Memphis, TN, USA)	302
IPT® (HOMS, Tokyo, Japan)	112
PFNA® (DePuy Synthes, Zuchwil, Switzerland)	109
Gamma3® (Stryker, Kalamazoo, MI, USA)	24
TFN-ADVANCED™ (DePuy Synthes)	23
Hansson DC Nail® (Swemac, Linköping, Sweden):	12
OM Short Femoral Nail® (OrthoMed, West Palm Beach, FL, USA)	6
CTC Nail® (KiSCO Co., Ltd., Kyoto, Japan)	4
Natural Nail® (Zimmer Biomet, Warsaw, IN, USA)	4
Unicorn Nail® (Hoya Technosurgical Co., Ltd., Tokyo, Japan)	2

These cephalomedullary nail implants were used in the study population. The table lists the brand names, manufacturers, and number of cases for each device. InterTAN®, PFNA®, and IPT® were the most commonly used implants.
