# Supplementary material for: Anterior Redisplacement After Intramedullary Nail Fixation for Trochanteric Femoral Fractures: Incidence and Risk Factors in 598 Older Patients
Source: J Clin Med. 2025 Aug 6;14(15):5557. doi: 10.3390/jcm14155557 (PMC12346959; doi:10.3390/jcm14155557)
Supplement: Supplementary file 1 [file jcm-14-05557-s001.zip › Supplementary_Table_S2.pdf]

**Table S2.** Preoperative and postoperative subtypes with the number and percentage of anterior redisplacement cases

| Preoperative Subtype | Postoperative Subtype | | |
|---|---|---|---|
| | **Posterior** | **Anatomical** | **Anterior** |
| **Posterior** | 59 (0, 0%) | 38 (6, 15.8%) | 12(0, 0%) |
| **Anatomical** | 35 (1, 2.9%) | 106 (11, 10.4%) | 10(0, 0%) |
| **Anterior** | 110（6, 5.4%） | 195 (49, 25.1%) | 33(0, 0%) |

This table shows the distribution of reduction subtypes from preoperative to postoperative radiographs. Values are presented as total cases (number of anterior redisplacements and percentage). Anterior redisplacement was defined as a shift of the distal fragment to the anterior position at follow-up. Cells showing “(0, 0%)” in the anterior column indicate that no further displacement is possible once the postoperative subtype is already anterior.
